# P-154. Endemic Diarrheal Pathogens during COVID-19 and Water Shortages: Analysis from Northeast Mexico

**DOI:** 10.1093/ofid/ofaf695.379

**Published:** 2026-01-11

**Authors:** José I Castillo, Sara P Rosales, Jorge A Vera, Susana P Cantú, Daniel Siller, Dzoara L Lugo, Cynthia D Peña, Héctor M Sánchez, Luis A Vásquez, Fátima Mellado, Bernardo A Fernández

**Affiliations:** Christus Muguerza Sistemas de Salud, S.A de C.V, Monterrey, Nuevo Leon, Mexico; Christus Muguerza Sistemas de Salud, S.A de C.V, Monterrey, Nuevo Leon, Mexico; Christus Muguerza Sistemas de Salud, S.A de C.V, Monterrey, Nuevo Leon, Mexico; Christus Muguerza Sistemas de Salud, S.A de C.V, Monterrey, Nuevo Leon, Mexico; Christus Muguerza Sistemas de Salud, S.A de C.V, Monterrey, Nuevo Leon, Mexico; Christus Muguerza Sistemas de Salud, S.A de C.V, Monterrey, Nuevo Leon, Mexico; Christus Muguerza Sistemas de Salud, S.A de C.V, Monterrey, Nuevo Leon, Mexico; Christus Muguerza Sistemas de Salud, S.A de C.V, Monterrey, Nuevo Leon, Mexico; Christus Muguerza Sistemas de Salud, S.A de C.V, Monterrey, Nuevo Leon, Mexico; Christus Muguerza Sistemas de Salud, S.A de C.V, Monterrey, Nuevo Leon, Mexico; Christus Muguerza Sistemas de Salud, S.A de C.V, Monterrey, Nuevo Leon, Mexico

## Abstract

**Background:**

Diarrheal diseases affect around 1.7 billion people annually. These diseases are preventable with access to safe water, hygiene, and sanitation. Environmental factors, such as seasonal droughts, can impact their incidence. During the pandemic, gastroenteritis cases decreased significantly. We aim to analyze the impact of the pandemic and water shortages on endemic diarrheal pathogens.

**Figure:**
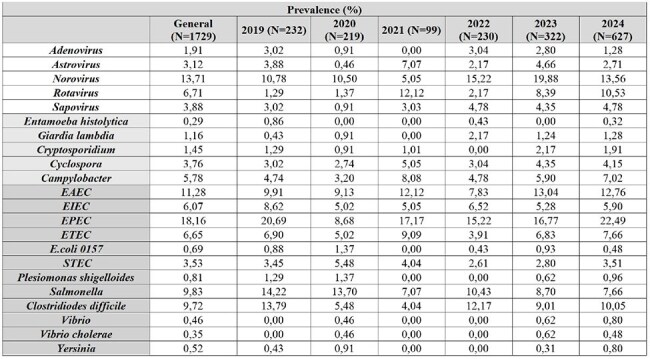
Trends in Gastrointestinal Pathogens Prevalence Over 6 Years of Study. Abbreviations: EAEC, Enteroaggregative Escherichia coli; EIEC, Enteroinvasive Escherichia coli; EPEC, Enteropathogenic Escherichia coli; ETEC, Enterotoxigenic Escherichia coli; STEC, Shiga toxin-producing Escherichia coli.

**Figure:**
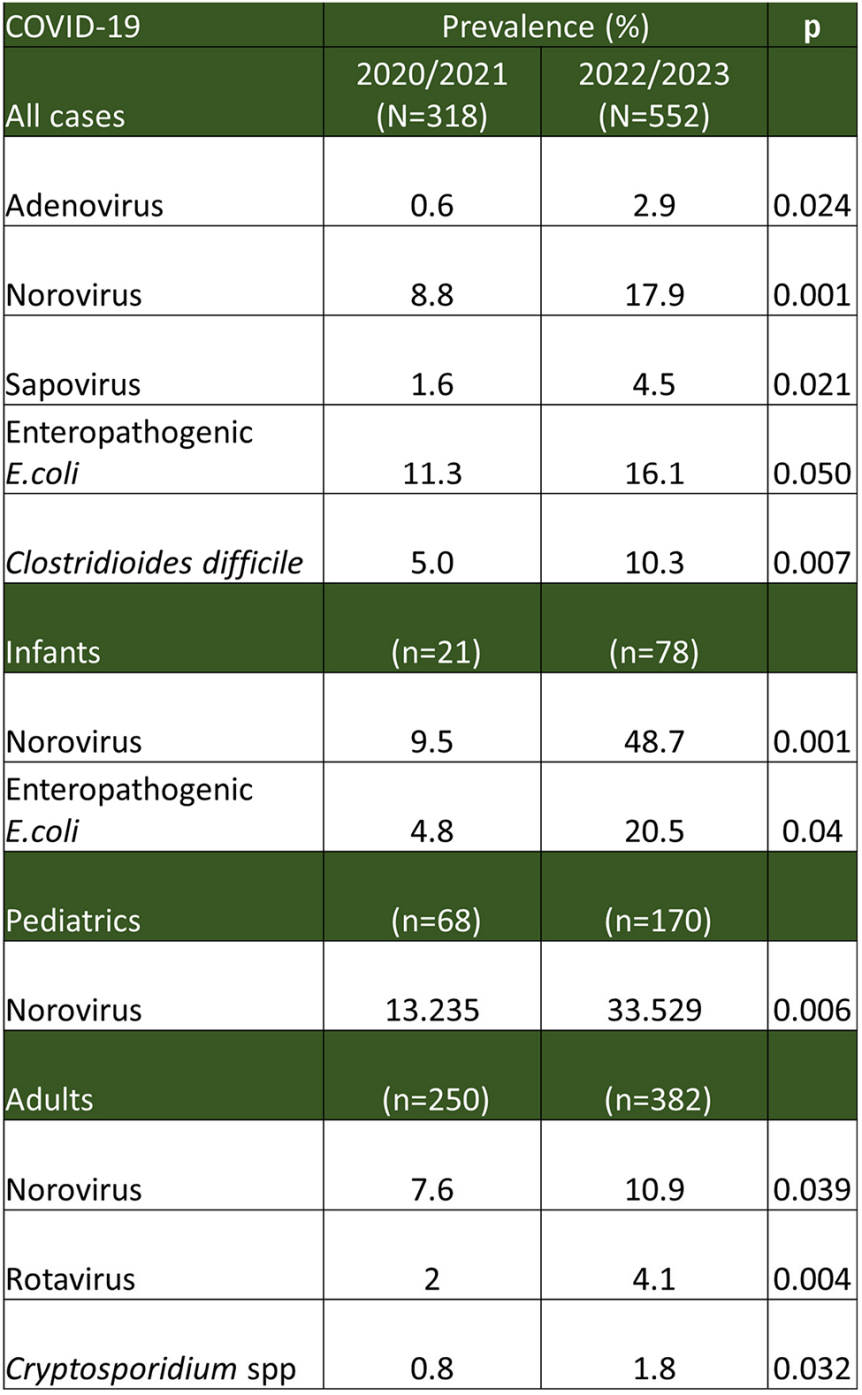
Comparison of Gastrointestinal Pathogen Frequencies and Prevalence in Pandemic Lockdown vs. Post-Lockdown Periods Across Age Categories. Age categories: Infants, <2 years; Pediatrics, <18 years; Adults, ≥18 years

**Methods:**

A retrospective study was conducted in Monterrey, Mexico, including hospitalized cases with acute diarrhea and available BIOFIRE® FILMARRAY® panel results from January 2019 to December 2024. Data were categorized into pandemic lockdown (2020-2021) and post-lockdown phases (2022-2023) and two water shortage periods (2019-2023 with shortages, 2022-2024 without). SPSS V27 was used for analysis, with a chi-square test for pathogen impact and Poisson regression for temporal distribution.

**Figure:**
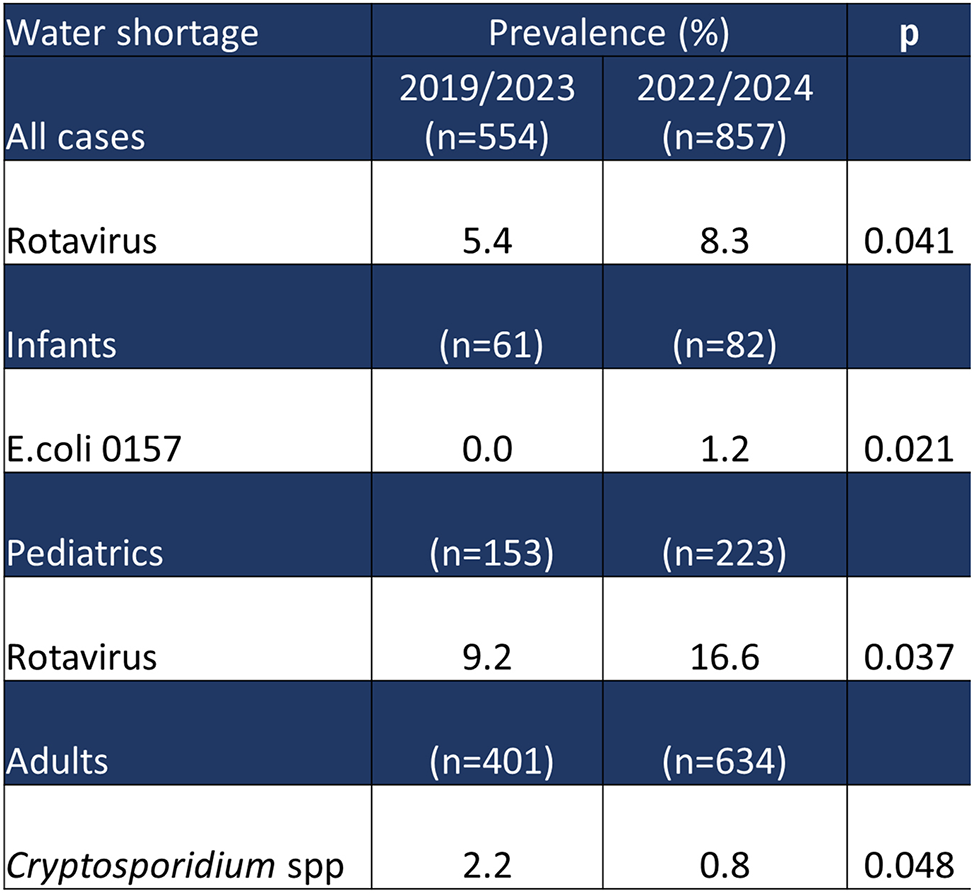
Comparison of Gastrointestinal Pathogen Frequencies and Prevalence with and without Water-Shortage Across Age Categories. Age categories: Infants, <2 years; Pediatrics, <18 years; Adults, ≥18 years

**Results:**

A total of 1,729 gastrointestinal panels were included. During the pandemic lockdown, viral pathogens decreased by 16.5%, bacterial pathogens by 6%, and parasites by 1.8%. Significant post-lockdown increase was seen in *Adenovirus* (0.63% vs. 2.9%; p=0.024), *Norovirus* (8.81% vs. 17.93%; p=0.000), *Sapovirus* (1.57% vs. 4.53%; p=0.021), *Enteropathogenic E. coli* (11.32% vs. 16.12%; p=0.050), and *C. difficile* (5.03% vs. 10.33%; p=0.007). Years without water shortages showed a 1% decrease in bacterial positivity and 1.1% in viral positivity. However, a significant increase in *Rotavirus* cases was observed during periods of water availability (5.4% vs. 8.28%; p=0.041).

**Conclusion:**

The pandemic lockdown led to a significant reduction in endemic diarrheal pathogens, likely due to non-pharmaceutical measures. Water shortages were associated with gastrointestinal outbreaks caused by viral and bacterial pathogens.

**Disclosures:**

All Authors: No reported disclosures

